# Retail food sales in Nunavut, Canada not impacted by short-term weather-related inaccessibility of trails used for Inuit subsistence harvesting

**DOI:** 10.1088/2976-601X/adf5c9

**Published:** 2025-09-25

**Authors:** Sappho Z Gilbert, James D Ford, Dylan G Clark, Lindsay Turner, Timothy O Fawehinmi, Amy Caughey, Shondra Stadnyk, Laurie Kaminsky, Rafael Pérez-Escamilla, Mahsa Jessri, Nicola L Hawley, Robert Dubrow

**Affiliations:** 1 Department of Nutrition, Harvard T.H. Chan School of Public Health, Boston, MA, United States of America; 2 Yale Center on Climate Change & Health, New Haven, CT, United States of America; 3 Priestley Centre for Climate Futures, University of Leeds, Leeds, United Kingdom; 4 Pacific Institute for Climate Solutions, Victoria, BC, Canada; 5 Department of Family Services, Government of Nunavut, Iqaluit, NU, Canada; 6 Department of Health, Government of Nunavut, Iqaluit, NU, Canada; 7 Nunavut Research Institute, Iqaluit, NU, Canada; 8 The North West Company, Winnipeg, MB, Canada; 9 Department of Social and Behavioral Sciences, Yale School of Public Health, New Haven, CT, United States of America; 10 Food, Nutrition and Health Program, Faculty of Land & Food Systems, University of British Columbia, Vancouver, BC, Canada; 11 Centre for Health Services and Policy Research, School of Population and Public Health, University of British Columbia, Vancouver, BC, Canada; 12 Department of Chronic Disease Epidemiology, Yale School of Public Health, New Haven, CT, United States of America; 13 Department of Environmental Health Sciences, Yale School of Public Health, New Haven, CT, United States of America

**Keywords:** weather, consumer behavior, Inuit, Canadian Arctic

## Abstract

Inuit have long utilized trail networks for subsistence harvest. Fueled by climate change, increasingly volatile environmental and weather conditions in the Canadian Arctic territory of Nunavut have made these routes less reliable and more dangerous—jeopardizing availability of traditional foods. Qualitative research indicates communities adapt by grocery shopping. We modeled consecutive days of trail inaccessibility on total grocery and meat product sales, respectively, of a market-dominant retailer in 13 Nunavut communities. Although we hypothesized positive associations between trail inaccessibility and store purchasing, we observed negligible negative associations; meanwhile, socioeconomic factors like pay dates yielded strong, positive associations. In light of the null findings with respect to trail inaccessibility, we discuss key limitations of our approach and potential alternative explanations that might account for these unexpected findings, including the ecological level of analysis potentially masking subgroup vulnerabilities relative to exposure or outcomes, hunters’ possible risk tolerance elasticities, and food sourced beyond our partner retailer (such as from the other major retail chain or through food sharing networks). Given the local nutrition and economic transitions—away from traditional food and subsistence livelihoods—communities may face reduced day-to-day vulnerability to trail accessibility disruptions. As the wage-based economy expands and the contemporary diet includes more energy-dense, processed store-bought food, Inuit communities may become increasingly sensitive to macro-political and economic pressures on their food system and sovereignty.

## 1. Introduction

**Figure 1.**
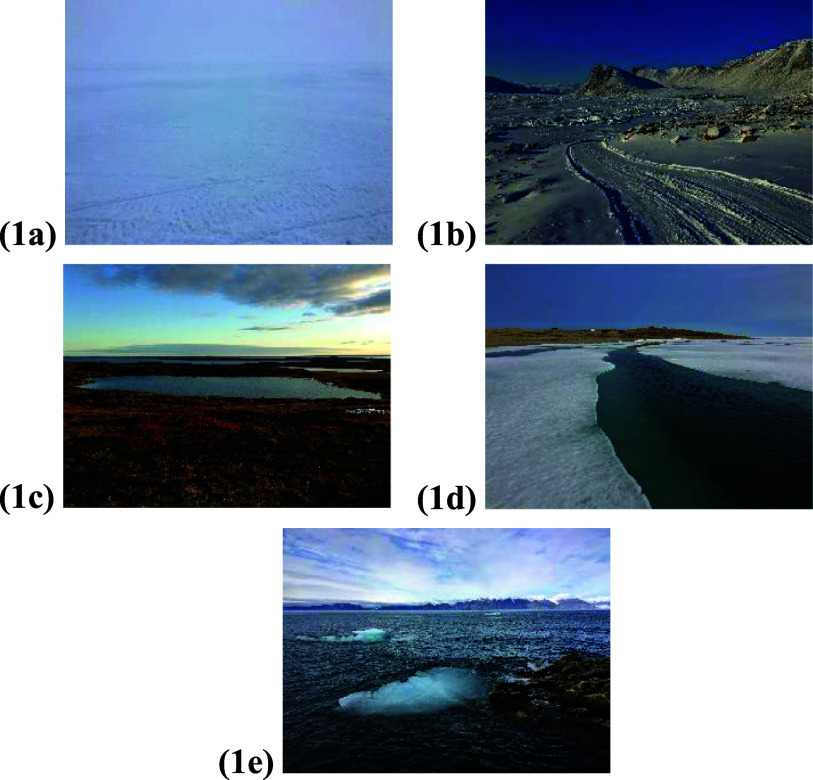
The three trail types (land, sea ice, and open water) in various Inuit communities and seasons: (a) sea ice with low visibility (near Iqaluit during Ukiuq; 8 March 2018); (b) land (Grise Fiord during Ukiuq; 15 March 2018); (c) land (near Rankin Inlet during Aujaq; 6 September 2015); (d) sea ice melting (near Cambridge Bay during Upingaaq; 8 July 2018); (e) open water (Pond Inlet during Aujaq; 3 August 2018). Photographs by Sappho Gilbert.

Since time immemorial, Inuit have harvested along land and sea (open water or ice, depending on the season) trail routes (figure 1) (Furgal and Seguin 2006, Ford 2009, Beaumier and Ford 2010, Statham *et al* 2015, Ford *et al* 2019). Country food is that which is hunted, trapped, whaled, fished, or picked, including caribou, narwhal, seal, muskox, Arctic char, and berries; these foods are nutrient-rich, often eaten raw or with minimal preparation, and remain a culturally meaningful part of the diet of many Inuit after over half a century of settled life (Kuhnlein *et al* 2004, Kuhnlein and Receveur 2007). With growing climatic and non-climatic threats to subsistence in the Canadian Arctic territory of Nunavut, store-bought food has increasingly supplanted country food in the present-day Inuit diet (Kenny *et al* 2018, Little *et al* 2021).

The circumpolar north is experiencing climate change at an accelerated rate relative to the rest of the planet—challenging expectations of seasons, availability of healthy flora and fauna for harvest, and other aspects of subsistence (Furgal and Seguin 2006, Ford 2009, Wakegijig *et al* 2013, Statham *et al* 2015). Across the Canadian Arctic, the safe use of trail networks for harvesting and other travel purposes has been jeopardized (Ford *et al* 2019). Routes have become more hazardous as multi-year sea ice is being replaced by less stable seasonal ice, sea ice coverage exists for shorter periods each year, and weather has become less predictable (Inuit Tapiriit Kanatami 2019). Many hunters are concerned about the resultant rising physical, economic, and cultural costs like vehicle and equipment damage, changing ecological knowledge, and declining harvests (Ford *et al* 2013, Pearce *et al* 2015).

Non-climatic pressures also have accelerated the local nutrition transition, or population dietary decrease in country food intake and increase in store-bought food intake (Drewnowski and Popkin 1997, Kuhnlein *et al* 2004). The public health nutrition impacts of this shift are substantial, largely owing to the high energy density and poor nutritional value of commonly purchased store-bought foods—which contributed over 70% of daily energy intake, per the 2007–2008 Inuit Health Survey (The Expert Panel on the State of Knowledge of Food Security in Northern Canada 2014)—as well as more sedentary lifestyles. Furthermore, among other diet-related chronic conditions, obesity and overweight have been rapidly rising in Nunavut this century (Kuhnlein *et al* 2004, Sheikh *et al* 2011, Government of Nunavut 2012, 2014, Statistics Canada 2014). While still important to communities, subsistence livelihoods have shrunk as the wage-based economy expands (Harder and Wenzel 2012, Statistics Canada 2018). Costly federal initiatives like the Nutrition North Canada program have centered retailers in the local food system—subsidizing their transport of eligible groceries to remote, Northern communities while investing relatively smaller amounts in support grants for harvesters (Ledrou and Gervais 2005, Tarasuk *et al* 2013, Arriagada 2017).

Despite these challenges, country food remains vital to Inuit culture and society. Numerous, primarily qualitative studies have highlighted an enduring desire for and harvest of country food, particularly in smaller communities (Chan *et al* 2006, Beaumier and Ford 2010, Wakegijig *et al* 2013, Statham *et al* 2015, Gilbert *et al* 2021). According to the 2017 Aboriginal Peoples Survey, 65% of Inuit surveyed in Nunavut reported hunting, fishing, or trapping in the previous 12 months (Government of Canada 2018b). A 2017–2018 Qikiqtani Inuit Association survey in six Baffin Island communities reported 26% of participants were ‘active or intensive [harvesters] (meaning that they do more than just day trips or weekends, and participate in all seasons)’ (Qikiqtani Inuit Association 2018). However, overall and especially among youth, harvesting has declined in Nunavut, with a rising role of only a few ‘super-harvesters’ who, as the most harvesting-engaged in their communities, tend to provide country food to numerous households (Priest and Usher 2004, Ford and Beaumier 2011, The Expert Panel on the State of Knowledge of Food Security in Northern Canada 2014).

In a prior qualitative study by a subset of our team, harvesters and Elders were interviewed in two Nunavut communities about determinants and effects of low-yield harvest periods (Gilbert *et al* 2021). Several participants mentioned that when environmental and weather hazards like poor sea ice conditions or extreme fog occur, they are unable to harvest; to adapt to this short-term absence of country food, they buy groceries, with some specifically noting meat purchases (i.e. beef or chicken) and others buying groceries ‘in general’ (Gilbert *et al* 2021).

In another study, a subset of our team developed a local model of trail inaccessibility (henceforth, ‘the trail access model’) (Ford *et al* 2019). With Canadian Arctic project partners, including Inuit harvesters, they defined and validated thresholds of precipitation, temperature, wind, visibility, ice concentration, and, for sea ice trails, ice thickness, that would render land, sea ice, or open water trails unsafe for use (Ford *et al* 2019). These variables have been and are expected to continue to be impacted by climate change, further imperiling country food in the Inuit food system and diet (Ford *et al* 2023). Therefore, it is important to examine the relationship between trail inaccessibility and grocery purchasing behavior to provide novel insights into food-related adaptive practices during weather and environmental extremes.

Using an ethnoclimatological approach, we joined large-scale population-level grocery sales data with the trail access model rooted in Inuit knowledge and environmental science. To our knowledge, this is the first published study of the relationship between weather-dependent trail access and store-bought food sales. Sales data from The North West Company (NWC), a market-dominant food retail chain in Nunavut, were acquired; these data overlapped with the trail access model for 13 Nunavut communities between 1 February 2013 and 31 May 2016.

Our aim was to investigate whether 1, 2, 3, or ⩾4 consecutive days of trail inaccessibility was associated with an increase in grocery spending overall and/or in sales volume of meat and meat products compared to days with at least one trail type safe for use. We anticipated spending on groceries overall and on sales volume of meat and meat products would increase on days of trail inaccessibility relative to days with at least one safe trail.

## 2. Methods

Data pre-processing, coding, and analysis were performed using Stata 17.0 SE (StataCorp LLC, College Station, TX, USA).

### 2.1. Grocery sales data & community deidentification

Beyond its academic and in-territory collaborations, this work was crucially partnered with NWC, one of only two major grocery chains present across the Canadian Arctic. The geographic and temporal coverage in common between the sales dataset provided by NWC and the trail access model encompassed the time period of 1 February 2013 to 31 May 2016 in 13 communities (figure S1 in supplementary online materials): Arviat, Baker Lake, Cambridge Bay, Clyde River, Coral Harbour, Gjoa Haven, Iqaluit, Kinngait, Kugluktuk, Pond Inlet, Rankin Inlet, Sanirajak, and Taloyoak. To protect community privacy, and at the request of in-territory project partners, we grouped communities other than the territorial capital of Iqaluit by population size—Large, Medium, and Small—and randomly assigned communities as A, B, C, and D within each.

Table 1 summarizes the means and standard deviations for key community characteristics by community grouping, with each characteristic also reported for Iqaluit. Due to its diverse demographics (approximately half of residents are non-Inuit), unique access to numerous food retail options in-town and online, and the largest population by far, Iqaluit is reported separately.

**Table 1.** Characteristics of Iqaluit and mean characteristics (with standard deviation) among groups of the other, deidentified 12 communities in this study (Naylor 2019, Statistics Canada 2019).

Group of communities^a^ or Iqaluit	Population (in 2016)	Median total income (Canadian $)	Median age (years)	Employment rate (%)	Mean number of food retailers^b^
**Iqaluit**	7740	70 695	31.1	73.9	4
**Large** (Arviat, Baker Lake, Cambridge Bay, Rankin Inlet)	2334 (502)	30 809 (9478)	25.4 (2.9)	54.4 (11.1)	2.5 (0.6)
**Medium** (Gjoa Haven, Kinngait, Kugluktuk, Pond Inlet)	1468 (121)	20 133 (1477)	23.8 (1.9)	44.3 (2.5)	2.0 (0.0)
**Small** (Clyde River, Coral Harbour, Sanirajak, Taloyoak)	955 (101)	21 653 (1092)	21.0 (0.7)	39.4 (4.2)	1.8 (0.5)

^a^ Ordering of the communities listed in parentheses is strictly alphabetical and does not necessarily correspond to the A, B, C, and D deidentified community names that have been randomly assigned within each group.

^b^ Number of food retailers (data largely derived from Naylor 2019). This variable includes in-town food retail stores; the territorial capital of Iqaluit has unrivaled access to numerous convenience stores, a gas station that sells snacks, and other options like online grocery stores and related retailers. As the exception, we manually set Clyde River’s value to 1 food retailer, as only an NWC store operated there during our study period.

For each community’s store, daily product-level aggregate sales data were provided by NWC as total sales volume (in grams) and as a daily percentage of annual sales dollars spent in-store by fiscal year (i.e. 1 February to 31 January). This latter metric, called ‘daily relative grocery expenditure’ throughout this paper, is in lieu of daily product-level expenditure in absolute dollar amounts, which NWC declined to provide.

### 2.2. Trail access model to determine days with no safe trail access

For the variable of harvesting trail inaccessibility nearby a community, we utilized the trail access model by Ford *et al* (2019). It is based on Environment and Climate Change Canada (ECCC) data on daily average temperature, total precipitation, average wind speed, and average visibility, as well as Canadian Ice Service (CIS) data on weekly average ice concentration and thickness (Ford *et al* 2019). Community-specific data from 1985 to 2016 and transcripts from interviews with Inuit harvesters (for each trail type and category of trail user risk tolerance: low, normal, high) were analyzed to develop model thresholds for daily inaccessibility status for each trail type (land [year-round], water [in open water seasons], and sea ice [during cold months; in other words, in reciprocal seasons to open water]) and risk tolerance level. A trail was deemed inaccessible on a particular date if >15% of the ECCC and CIS parameters were beyond the limits of safe access for that kind of trail; for instance, visibility under 1 kilometer on a land trail would be considered a threshold failure (Ford *et al* 2019). The thresholds were designed with and approved by Inuit community members and a team of academic researchers ‘with a combined 135 years of experience working with Inuit communities’ (Ford *et al* 2019).

For the purposes of this study, a safe day for trail use was defined as having at least one trail type accessible for a normal-risk trail user: either a land or water trail (in open-water seasons) or a land or ice trail (in sea ice seasons). Normal-risk tolerant individuals were defined as lifelong trail users ‘with some experience in stressful situations while traveling and [who] can make a shelter,’ while high-risk tolerant individuals possess ‘an in-depth understanding of how trail conditions are affected by climate-related conditions, knowledge of alternative routes, and well-developed skillsets’ and low-risk tolerant users generally have less training and/or experience to confidently travel ‘in a broader set of conditions’ (Ford *et al* 2019). The phenomenon of ‘trail switching,’ as described in the literature, occurs when a harvester decides to use a different type of trail due to their preferred one being inaccessible (often due to environmental and weather issues); as such, we designed our main independent variable of interest—trail inaccessibility—to be 1, 2, 3, or 4 or more (⩾4) consecutive days in which neither land nor water trails (in open-water seasons) or neither land nor ice trails (in sea ice seasons) were safe for normal-risk users. There were no days where water and sea ice trails were both accessible. The majority of communities had data available from the trail access model for our entire period of study (1216 days). Five had slightly fewer days with data available: Baker Lake (1204), Cambridge Bay (1214), Clyde River (1185), Coral Harbour (1211), and Sanirajak (1208). Trail status for dates with missing data was approximated using linear splines (as used by Ford *et al*), resulting in 1216 days of complete trail access data for all communities.

### 2.3. Estimating date ranges for the 6 Inuit seasons

Inuit enjoy six seasons each year, the dates of which are specific to each subregion in the territory (table S1 in supplementary online materials) as per consultation with communities and harvesters in the drafting of the Nunavut Land Use Plan’s (NLUP) (Nunavut Planning Commission 2016, 2021). Although they are called ‘regions’ in the NLUP, we have termed them subregions in order to avoid confusion with the territory’s three main administrative regions. In the four coldest Inuit-defined seasons—Ukiaq, Ukiuq, Upingaksaaq, and Upingaaq—sea ice trails are used in addition to year-round land trails. The seasons of Aujaq and Ukiaqsaaq, which largely align with late summer and fall in the four-season calendar, are generally considered open-water seasons during which water trails are accessible (in addition to land).

Due to variability in exact sea ice break-up dates by community (even within a single subregion), we utilized community-specific shorefast ice break-up dates to define the beginning of Aujaq (table S1 in supplementary online materials) instead of the dates listed in the NLUP. This approach enhanced the precision of our demarcated start dates of open water versus sea ice for trail use. After consulting our in-territory collaborators about when open water would be perceived as safe for typical harvesters, we decided to use community dates when ⩾90% short-fast ice break-up was typically reached this century, which are also readily available in the literature (Bell and Brown 2018, Cooley *et al* 2020). For 11 of the communities, data were derived from Cooley *et al*’s analysis of these break-up dates between 2000 to 2018 (2020). Two of our communities of study—Baker Lake and Sanirajak—did not have data in Cooley *et al*; these break-up dates were instead estimated based on CIS’ 30 year Climatic Ice Atlas for 1991–2020 as well as the 1981–2010 average ice break-up dates from ArcticNet’s Integrated Regional Impact Study (Bell and Brown 2018, Canadian Ice Service 2021). Table 2 summarizes this approach.

**Table 2.** Date ranges within a calendar year used to determine which two trail types were required to meet the definition of daily trail inacessibility.

	Required to be inaccessible in order meet definition of daily trail inaccessibility		
Date range	Land	Sea ice	Open water
January 1 to the day of year of ⩾90% shorefast ice breakup^b^ in community	Yes	Yes	N/A^a^
Day of year of ⩾90% shorefast ice breakup^b^ to the start of Ukiaq	Yes	N/A^a^	Yes
Start of Ukiaq to December 31	Yes	Yes	N/A^a^

^a^ N/A denotes not applicable and not used in trail inaccessibility determination for that date range. No two dates for any community in the dataset have sea ice and open water simultaneously accessible.

^b^ Numbered day of the non-leap year at which ⩾90% shorefast ice breakup can be expected: Arviat = 182 (1 July), Baker Lake = 186 (5 July), Cambridge Bay = 207 (26 July), Clyde River = 208 (27 July), Coral Harbour = 188 (7 July), Gjoa Haven = 210 (29 July), Iqaluit = 184 (3 July), Kinngait = 184 (3 July), Kugluktuk = 187 (6 July), Pond Inlet = 205 (24 July), Rankin Inlet = 186 (5 July), Sanirajak = 145 (25 May), and Taloyoak = 211 (30 July).

### 2.4. Statistical analyses

Our primary independent variable was community-specific, daily trail inaccessibility according to the number of consecutive inaccessible days: 0 (accessible), 1 (first day of inaccessibility), 2 (second), 3 (third), ⩾4 (fourth or greater). The primary outcome variables were daily relative grocery expenditure and sales of meat and meat products, tabulated each day for each community NWC store. Meat and meat products include items categorized as Fresh Meat, Frozen Meat, or Processed Meats in our sales dataset. Daily relative grocery expenditure was the percent of fiscal year annual sales dollars spent at the store by the community on all groceries (including non-food/beverage items) on that particular day. Daily sales of meat and meat products was expressed as grams (g) per capita; for each day, we divided grams sold by the community population size in that year (as estimated on an annual basis via linear interpolation between Census 2011 and 2021 data).

Both outcome variables exhibited right-skewed non-normality. In general, outliers above the 99th percentile and below the 1st percentile were unrelated to trail inaccessibility, month, or other key variables. These outliers are very likely related to factors such as large community events that served food and beverages or could be simply spurious data due to entry errors that were later corrected as refunds. Outliers below the 1st percentile were often in holiday periods when there may have been reduced store hours; there may have been non-holiday days with reduced hours in this lower end of our outcome distributions as well. To mitigate the influence of extreme values, we winsorized each community’s two outcome variables below the 1st and above the 99th percentiles (Tukey 1962). Although winsorization improved normality of each community’s dependent variable distribution by visual inspection, non-normality persisted according to Shapiro–Wilk tests.

To examine whether trail inaccessibility yielded statistically significantly different mean outcomes for our two outcome variables in each community, we conducted two-sample t-tests for each outcome. We also reported the number of days (and their percent of total study period days) by trail inaccessibility status for each community.

To test the relationship between consecutive days of trail inaccessibility and total grocery and meat/meat product sales, we used multivariable Poisson regression models with a Huber/White/Sandwich linearized estimator of variance; this estimator returns robust standard errors and permits the violation of the usual Poisson assumption of an equal mean and variance (Nichols 2010, Wooldridge 2010). In each model, we included the following covariates: Inuit season; day of week; fiscal year (for daily relative grocery expenditure; between 1 February and 31 January) or calendar year (for meat/meat product sales volume); whether or not it was a pay date for the Government of Nunavut (GN), which employs roughly 57% of Inuit in the territory; whether or not it was a Canada Child Benefit payment date (a tax-free monthly payment from the Canada Revenue Agency to families with children under 18 years old); and if the date fell on a federal or territorial holiday or the full period between Christmas Eve and New Year’s Day, inclusive (Government of Canada 2018a). GN pay dates, federal and territorial holidays, and Canada Child Benefit dates were provided by Nunavut-based co-authors of this project. Reference categories for our covariates were: Day 0 for trail inaccessibility (in other words, safe days), Ukiuq for Inuit-defined season, Monday for day of week, 2013 for fiscal year (for daily relative grocery expenditure) and year (for daily meat/meat product sales volume), not a GN pay date, not a Canada Child Benefit payment date, and not a holiday. Our definition of holiday to include the days between Christmas and New Year’s Day aligns with other population nutrition research using grocery sales data (Petimar *et al* 2022). For each of the two outcome variables, we ran a separate model for each community. To ease interpretation, we exponentiated Poisson model coefficients and their 95% confidence limits to produce a daily relative grocery expenditure ratio, a daily meat and meat products sales volume purchase ratio, and similar ratios for each covariate. For each variable, these ratios were then converted to percent change (compared to the reference category) for ease of interpretation.

For each outcome variable, we then conducted random effects meta-analyses across the communities to estimate pooled effects for consecutive day of trail inaccessibility and for each covariate. We used a restricted maximum likelihood approach in our random-effects meta-analyses to take into account the loss in degrees of freedom resulting from the pooled effect estimation and to mitigate the downward bias of the between-study variance estimator (Kontopantelis and Reeves 2010). Between-community heterogeneity was assessed using the I^2^ statistic and the Q-statistic with the associated chi-squared test and *p*-value.

## 3. Results

In our 40 month study period, all 13 communities had access to at least one safe trail most days (table S2 in supplementary online materials). Percentage of trail-inaccessible days by community ranged from 5% to 24%, with a rough north-to-south gradient of increasing trail inaccessibility. Overall, meaningful community-specific differences were not observed in total grocery or meat/meat product sales between inaccessible versus accessible trail days (table S2 in supplementary online materials).

**Figure 2.**
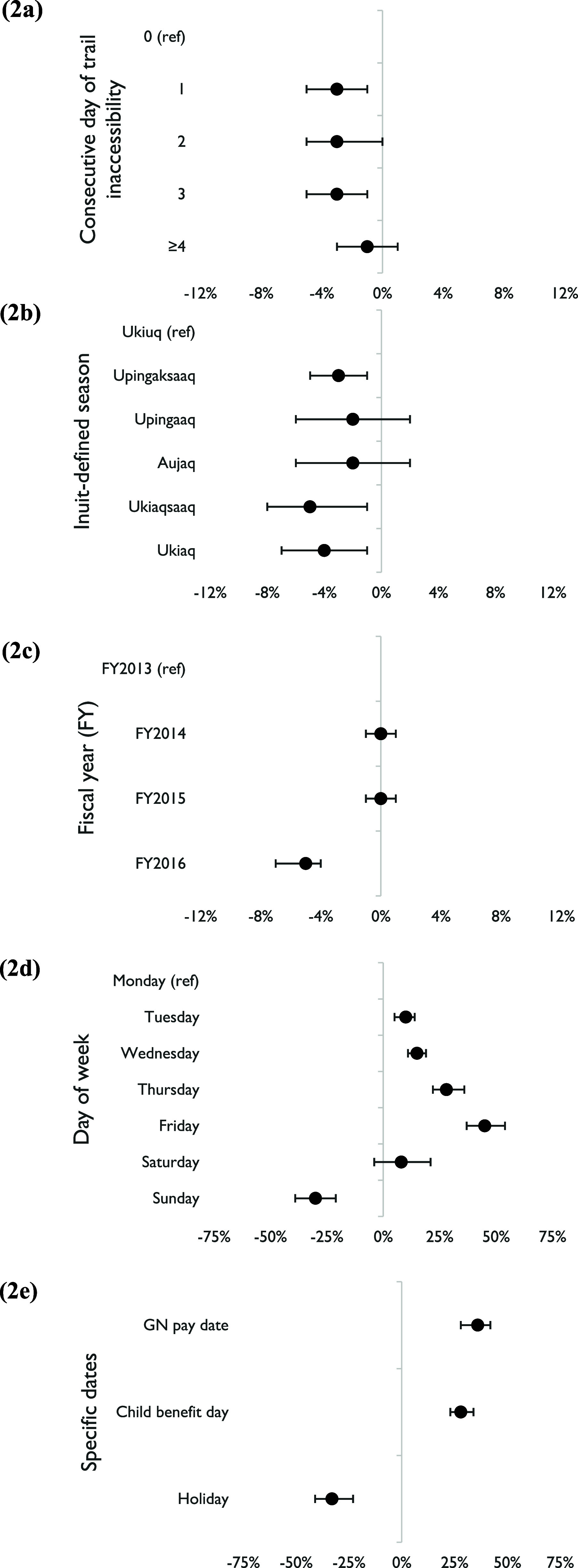
Pooled estimates (with 95% confidence interval) across all 13 study communities of percent change in daily relative grocery expenditure (%) for each model variable: (a) consecutive day of trail inaccessibility; (b) Inuit-defined season; (c) fiscal year; (d) day of week; (e) specific dates. N.B.: GN = Government of Nunavut; ‘Child benefit day’ = monthly Canada Child Benefit date.

Figure 2 (along with table S3 in supplementary online materials) presents pooled estimates across communities’ estimates of the adjusted percent change in daily relative grocery expenditure (as a percent of annual sales dollars spent at the store) for 1, 2, 3, and ⩾4 consecutive days of trail inaccessibility compared to days when trails were accessible. We observed statistically significant decreases of 3% on Day 1 (95% CI: −5%, −1%) and Day 3 (−5%, −1%), with low to moderate inter-community heterogeneity. Neither Day 2 nor Day ⩾4 revealed significant change.

Regarding covariates, pooled estimates for three Inuit seasons were significantly lower than for Ukiuq (reference season): Upingaksaaq (−3% [−5%, −1%]), Ukiaqsaaq (−5% [−8%, −1%]), and Ukiaq (−4% [−7%, −1%]). Daily relative grocery expenditure was significantly lower in fiscal year 2016 versus fiscal year 2013 (−5% [−7%, −4%]). Relative to Monday, we observed a significant change in daily relative grocery expenditure for every day of the week except Saturday: increases on Tuesday (10% [5%, 14%]), Wednesday (15% [11%, 19%]), Thursday (28% [22%, 36%]), and Friday (45% [37%, 54%]), with a decrease on Sunday (−30% [−39%, −21%]). GN pay date (36% [28%, 42%]) and monthly Canada Child Benefit day (28% [23%, 34%]) showed significant increases in daily relative grocery expenditure compared to other days, while holidays exhibited a decrease relative to non-holiday dates (−33% [−41%, −23%]).

Community-specific results for daily relative grocery expenditure are shown in table S3 (supplementary online materials). We observed significant decreases in daily relative grocery expenditure in two communities: Large B on Days 1 (−7% [−10%, −3%]) and ⩾4 (−5% [−9%, −2%]), and Small C on Day 3 (−15% [−29%, −1%]). In all communities, daily relative grocery expenditure was significantly higher on Thursday and Friday relative to Monday, GN pay dates versus non-pay dates, and monthly Canada Child Benefit days compared to other days. Expenditure was consistently higher on Tuesday (8 communities statistically significant; 1 non-significantly lower) and Wednesday (11 significant) but consistently lower on holidays versus other days (12 significant), on Sunday (relative to Monday; 11 significantly lower; 1 significantly higher), and during fiscal year 2016 versus 2013 (8 significantly lower). There were less consistent and fewer significant community-specific estimates for Inuit-defined seasons.

**Figure 3.**
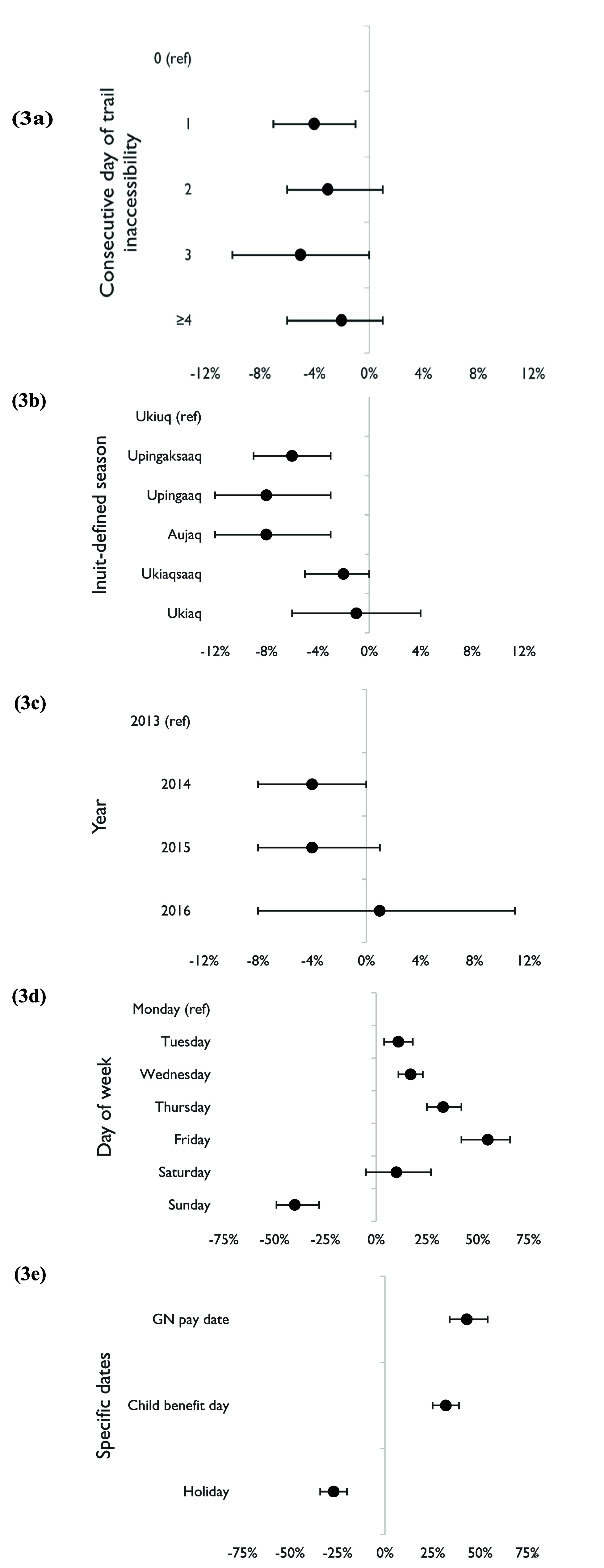
Pooled estimates (with 95% confidence interval) across all 13 study communities of percent change in meat and meat product sales volume (grams per capita) for each model variable: (a) consecutive day of trail inaccessibility; (b) Inuit-defined season; (c) year; (d) day of week; (e) specific dates. N.B.: GN = Government of Nunavut; ‘Child benefit day’ = monthly Canada Child Benefit date.

Figure 3 (along with table S4 in supplementary online materials) shows pooled estimates for percent change in daily sales of meat and meat products (grams per capita) on trail-inaccessible days compared to trail-accessible days. The only significant change was a decrease on Day 1: −4% (−7%, −1%), with moderate heterogeneity. Compared with Ukiuq, we observed significant decreases in pooled estimates for three Inuit seasons: Upingaksaaq (−6% [−9%, −3%]), Upingaaq (−8% [−12%, −3%]), and Aujaq (−8% [−12%, −3%]). Compared to 2013, none of the subsequent three years yielded a significant percent change. Similar to daily relative grocery expenditure, we observed significant changes in meat and meat product sales for GN pay date (43% [34%, 54%]), Canada Child Benefit day (32% [25%, 39%]), holidays (−27% [−34%, −20%]), and non-Saturday days of week relative to Monday: Tuesday (11% [4%, 18%]), Wednesday (17% [11%, 23%]), Thursday (33% [25%, 42%]), Friday (55% [42%, 66%]), and Sunday (−40% [−49%, −28%]).

Table S4 in the supplementary online materials presents community-specific results for meat and meat product sales. Five communities had significant decreases on inaccessible versus accessible trail days: Day 1 in Large B (−10% [−15%, −4%]) and Large D (−11% [−19%, −4%]); Day 2 in Large A (−8% [−15%, −1%]), Day 3 in Small C (−20% [−33%, −7%]) and Small D (−28% [−53%, −4%]), and Day 4 in Large B (−9% [−15%, −4%]), and Large D (−9% [−16%, −2%]). Like with daily relative grocery expenditure, all 13 communities exhibited significantly elevated sales of meat and meat products on Thursday and Friday (versus Monday), on GN pay dates (versus non-pay dates), and on monthly Canada Child Benefit days (versus other days). Once again, sales were consistently higher on Tuesday (7 significantly higher; 1 significantly lower) and Wednesday (10 significant) relative to Monday, but were consistently lower on holidays compared to non-holidays (9 significant) and Sunday (relative to Monday; 11 significantly lower; 1 significantly higher). For Inuit-defined seasons and year, there were fewer and less consistent significant community-specific estimates.

## 4. Discussion

We hypothesized that consecutive days of trail inaccessibility in 13 Nunavut communities would predict significantly more daily relative grocery expenditure and more daily sales volume of meat and meat products. However, our pooled estimates revealed statistically significant declines in each of the two outcomes on certain consecutive days of trail inaccessibility, with no significant increases. Significant community-specific results were also in the opposite direction of our hypothesis. For each outcome, significant community-specific declines were rare, relatively small, and not sustained over the consecutive trail-inaccessible days. Furthermore, there was no geographic or community population size-related pattern to these results.

Our findings suggest low day-to-day dependency on subsistence in our communities of study. Contrary to dietary surveys in Nunavut that have quantitatively tracked the displacement of country food by store-bought food (Sharma *et al* 2010, Sheikh *et al* 2011, Sheehy *et al* 2013, Kenny *et al* 2018), qualitative studies have tended to center country food in the Inuit food system (Chan *et al* 2006, Beaumier and Ford 2010, Ford and Beaumier 2011, Wakegijig *et al* 2013, Statham *et al* 2015, Gilbert *et al* 2021). Our hypotheses perhaps relied too heavily on communities’ qualitative emphases on the eminence of country food in their lives and diet and on the adaptive practice of grocery shopping when harvesting is not possible (Statham *et al* 2015, Gilbert *et al* 2021).

Our results align with the quantitated reality that, per the most recent territorially representative survey in 2007–2008, Inuit in the Canadian Arctic source over 70% of their daily caloric intake from store-bought food (Sheikh *et al* 2011, The Expert Panel on the State of Knowledge of Food Security in Northern Canada 2014), in contrast to the millennia-long dietary pattern wherein country food dominated due to semi-nomadic life and continual harvest with near-immediate consumption (Wenzel 2000, Kuhnlein *et al* 2004, Wenzel 2013). Nevertheless, country food retains irreplaceable cultural and economic value, and its shrinking dietary role bears negative implications for population nutrition and health (Kuhnlein *et al* 2004, Ford and Berrang-Ford 2009, Wakegijig *et al* 2013, Warltier *et al* 2021). As such, harvesting and country food should continue to be studied alongside communities with mixed methodologies to generate comprehensive, rigorous evidence in support of Inuit food sovereignty and well-being.

The nutrition transition in Nunavut has occurred in a two-way, positive feedback loop within the broader, ongoing food system transformation and a legacy of colonization in Canada’s North: store-bought food necessitates income, deepening trends away from subsistence livelihoods and toward the wage-based economy, further elevating store-bought food over country food (Kuhnlein *et al* 2004, Sharma *et al* 2010, Ford and Beaumier 2011, Harder and Wenzel 2012). Moreover, equipment, gasoline, and other costs associated with harvesting have risen over the years, pushing some away from harvesting and kindling a continual need for income—whether for groceries or harvesting—in this high-poverty population (Wenzel 2013). Income (more accurately, money) is now a hinge point of this evolving food system across Inuit communities: those with higher income tend to be able to afford more store-bought food and/or harvesting equipment (Harder and Wenzel 2012, Ready 2016, Hillemann *et al* 2023). However, these same individuals tend to be engaged in wage labor that leaves them less time available for harvesting; meanwhile, those not employed in wage jobs have more time but ‘are cash-poor and thus limited in their ability to hunt’ (Wenzel 2017).

In view of this centrality of money in-flow in this mixed, contemporary food system, we included several financial variables in our model, which ended up exhibiting the largest effect sizes of all. Across all 13 communities, each of the covariates GN pay date, monthly Canada Child Benefit day, and Thursday or Friday (versus Monday) was associated with large, significant increases in total grocery sales and meat and meat product sales. Pay date of a prominent local employer (i.e. the GN) and days later in the work week (locally corresponding to pay dates of various non-GN income sources in Nunavut) are useful markers of a wage-based economic system. Consistent, strong impact on Canada Child Benefit day sales points to the high territorial prevalence of poverty and food insecurity (Statham 2012, Battle and Torjman 2013, Wakegijig *et al* 2013).

Strikingly, the relative increase in daily meat and meat product sales volume eclipsed that observed for daily total grocery expenditure for each of these economically relevant covariates. This suggests that, when Nunavut residents receive income, they not only buy more groceries in general than usual; in particular, they buy more meat and meat products. This aligns with the known Inuit preference for meat—unsurprising given traditional dietary patterns—and echoes the sentiment in the communities that, even with federal subsidies, such items tend to be economically out-of-reach.

Our work also quantitatively evinces what Statham once posited: ‘greater fondness for store foods’ would leave communities ‘less sensitive to climatic extremes’ (Statham 2012). Perhaps if our analysis had been run a few decades prior, we would have observed our anticipated store-bought food sales responses to short-term trail inaccessibility. While communities’ steady reliance on store-bought food may boost their resilience to climatic threats to trails used for harvest, it also leaves them more vulnerable to macro-stresses on their evolving, remote, Indigenous-majority food system—with potential detrimental effects on health and culture. These stresses include long and fragile food supply chains, national and global economic forces (such as inflation), federally led food policy for the territory (like the Nutrition North Canada program subsidizing retailers’ shipment of groceries to Northern communities), and reliance on government assistance programs like the monthly Canada Child Benefit. Climate change can disrupt timetables for flights and sealift (with changing, extending open-water seasons). While these changes are not all necessarily negative for food security, they do, on the whole, endanger food sovereignty.

Ford *et al* recently published a projection version of their model, estimating sea ice trail-inaccessible days will substantially increase by century’s end across 53 Inuit communities (including in Nunavut) (2023). They also project land trail-inaccessible days will decrease in Nunavut’s Baffin region and grow in the Kivalliq region (at both high and low greenhouse gas emissions scenarios), with the Kitikmeot region gaining land trail-inaccessible days in the low emissions scenario but losing them in the high emissions one. Growing periods with no safe access to trails of any type were also projected (Ford *et al* 2023). Although results of our present study found small to no short-term declines in daily total grocery expenditure or meat/meat product sales volume, longer stretches with high prevalence of trail-inaccessible days (as long, consecutive periods are relatively rare) should be studied for possible association with increased grocery sales.

A core strength of our study was the collaborative pairing of Inuit knowledge (via the hunter-validated trail access model and our use of the NLUP Inuit-defined seasonal dates) with scientific data (e.g. sea ice break-up dates) and analytic techniques. As Nunavut lacks a paved road system, communities exist as effectively closed food systems, minimizing spillover risk. Nunavut residents tend to shop more frequently and in smaller quantities compared to non-Arctic populations, meaning behavioral responses to stimuli (such as trail inaccessibility) are more likely detectable.

It is also important to consider study limitations and potential alternative explanations for our unanticipated results. One limitation to using grocery sales data is risk of ecological fallacy; there doubtlessly exist broad distributions of sensitivity to the cascading impacts of trail inaccessibility within each community. Some subpopulations who are highly reliant on trail accessibility for their food security may need to turn to store-bought food during times of trail inaccessibility, whereas other highly reliant subpopulations may not turn to store-bought food because they have a well-stocked refrigerator/freezer and/or stable access to a food sharing network. Relatedly, our data cannot distinguish Inuit from non-Inuit shoppers, who may each face distinct challenges and opportunities. This is particularly relevant in Iqaluit, where Inuit comprise roughly half the community, while all other communities in Nunavut are much higher proportion Inuit (Statistics Canada 2024).

Conceivably, environmental and weather conditions precluding safe trail use may also impede grocery shopping in the short term; if conditions are prolonged, supply chain disruptions due to cargo flight delays or cancellations may impact store stock and, thereby, sales. Trail access model thresholds were designed to be applicable across Canadian Arctic regions rather than for each community (Ford *et al* 2019). With respect to land trail use, the model does not capture: (1) whether there is enough snowpack for harvesters to use skidoos (versus all-terrain vehicles [ATVs]), and (2) in the summer, if trails are too muddy for ATV use. The model also does not incorporate the state of lake or river ice. Given the long history of Inuit harvesters adapting to environmental and weather challenges, misclassification of daily trail status is possible.

Our approach offered a novel yet partial view into a complex and evolving food system, as we were unable to account for non-NWC food sources (e.g. the second major grocery chain) or other adaptive behaviors, like use of food sharing networks or even simply going hungry (as described in some literature) (Beaumier and Ford 2010, Statham *et al* 2015). Nevertheless, NWC’s market dominance and largely consistent patterns observed across communities bolster confidence in our findings.

We initially considered using Ford *et al*’s high-risk trail user thresholds for maximal degree of trail inaccessibility for our exposure variable but found that the proportion of days with complete trail inaccessibility in our study period using this higher threshold was extremely limited. We therefore relied solely on the normal-risk trail user thresholds, and thereby we were unable to study risk tolerance elasticities (e.g. normal versus high thresholds). Interviews with Inuit hunters in Nunavut and the Northwest Territories have found some experienced harvesters resort to ‘risking it’ in poor conditions (Ford 2008, Naylor *et al* 2021). Perhaps the relatively few, ultra-engaged ‘super-harvesters’ who disproportionately account for country food acquisition and distribution today tend to be more high-risk tolerant or risk-elastic trail users, rendering it possible that harvesting persisted on days we classified as trail inaccessible. It also is possible that normal-risk trail users, in the face of high desire and need for country food, might be willing to temporarily take on a higher risk tolerance. However, harvesting activities have been declining in Nunavut for decades (especially among youth) (The Expert Panel on the State of Knowledge of Food Security in Northern Canada 2014). Thus, in addition to there being few trail inaccessible days in the high-risk tolerant model, there are likely ever-fewer such high-risk tolerant trail-using harvesters and more normal-risk or low-risk trail users.

Our study has three final limitations that are important to note. First, we conducted multiple comparisons for our primary predictor variables; with 13 communities and 4 inaccessible trail day levels, we conducted 52 comparisons. Therefore, some of our statistically significant community-specific results may be due to chance alone. Our pooled estimates were not subject to this limitation. Second, our daily relative grocery expenditure metric included some non-food/beverage items as well as food and beverages. However, the vast majority of items in our grocery sales dataset are, in fact, food and beverages. Furthermore, the consistency of results between this metric and the meat/meat products metric suggests the presence of a small proportion of non-food/beverage items did not bias our results. Finally, the trail access model was developed with Inuit harvesters to identify thresholds for conditions of safe travel on land, sea ice, and water routes as commonly used for hunting, fishing, and gathering beyond one’s community; it is not harvesting type-specific and does not account for all possible harvesting activities. It would require extensive additional engagement with Inuit harvesters to identify thresholds specific to more niche (and intra-community) activities like foraging, berrypicking, trapping, and more, as well as to map all of those potential trail networks.

Our study found that daily grocery expenditure and sales of meat and meat products were overall unaffected by 1, 2, 3, or ⩾4 consecutive days of trail inaccessibility in 13 Nunavut communities. In view of relevant literature, this suggests the local nutrition transition has progressed to where day-to-day reliance on travel for country food harvest has likely substantially receded. We also identified study limitations and potential alternative explanations for our unanticipated results that warrant further study. Future research and decision-making related to the Inuit food system should be led by communities’ values, needs, and desires. This is particularly salient given the subsistence economy’s fragility relative to the wage-based economy, which has received weighty investments like food retailer subsidies that outsize federal and other grants directly supporting harvesters.

## Data Availability

Restrictions apply. Grocery sales data were made available by The North West Company for this study via a data use agreement with Yale University. Along with the corresponding code, these data cannot be shared.

## References

[erfsadf5c9bib1] Arriagada P (2017). Food Insecurity Among Inuit Living in Inuit Nunangat. https://www150.statcan.gc.ca/n1/pub/75-006-x/2017001/article/14774-eng.htm.

[erfsadf5c9bib2] Battle K, Torjman S (2013). Poverty and Prosperity in Nunavut. https://assembly.nu.ca/library/GNedocs/2013/001057-e.pdf.

[erfsadf5c9bib3] Beaumier M C, Ford J D (2010). Food insecurity among Inuit women exacerbated by socioeconomic stresses and climate change. Can. J. Public Health.

[erfsadf5c9bib4] Bell T, Brown T M (2018). From Science to Policy in the Eastern Canadian Arctic: An Integrated Regional Impact Study (IRIS) of Climate Change and Modernization.

[erfsadf5c9bib5] Canadian Ice Service (2021). 30-Year Ice Climate Normals. https://iceweb1.cis.ec.gc.ca/30Atlas/page1.xhtml?lang=en.

[erfsadf5c9bib6] Chan H M, Fediuk K, Hamilton S, Rostas L, Caughey A, Kuhnlein H, Egeland G, Loring E (2006). Food security in Nunavut, Canada: barriers and recommendations. Int. J. Circumpolar Health.

[erfsadf5c9bib7] Cooley S W, Ryan J C, Smith L C, Horvat C, Pearson B, Dale B, Lynch A H (2020). Coldest Canadian Arctic communities face greatest reductions in shorefast sea ice. Nat. Clim. Change.

[erfsadf5c9bib8] Drewnowski A, Popkin B M (1997). The nutrition transition: new trends in the global diet. Nutr. Rev..

[erfsadf5c9bib9] Ford J D (2008). Climate, society, and natural hazards: changing hazard exposure in two Nunavut communities. Northern Rev..

[erfsadf5c9bib10] Ford J D (2009). Vulnerability of Inuit food systems to food insecurity as a consequence of climate change: a case study from Igloolik, Nunavut. Reg. Environ. Change.

[erfsadf5c9bib11] Ford J D, Beaumier M (2011). Feeding the family during times of stress: experience and determinants of food insecurity in an Inuit community. Geogr. J..

[erfsadf5c9bib12] Ford J D, Berrang-Ford L (2009). Food security in Igloolik, Nunavut: an exploratory study. Polar Rec..

[erfsadf5c9bib13] Ford J D, Clark D G, Copland L, Pearce T, Harper S L (2023). Projected decrease in trail access in the Arctic. Commun. Earth Environ..

[erfsadf5c9bib14] Ford J D, Clark D, Pearce T, Berrang-Ford L, Copland L, Dawson J, New M, Harper S L (2019). Changing access to ice, land and water in Arctic communities. Nat. Clim. Change.

[erfsadf5c9bib15] Ford J D, McDowell G, Shirley J, Pitre M, Siewierski R, Gough W, Duerden F, Pearce T, Adams P, Statham S (2013). The dynamic multiscale nature of climate change vulnerability: an Inuit harvesting example. Ann. Assoc. Am. Geogr..

[erfsadf5c9bib16] Furgal C, Seguin J (2006). Climate change, health, and vulnerability in Canadian northern Aboriginal communities. Environ. Health Perspect..

[erfsadf5c9bib17] Gilbert S Z, Walsh D E, Levy S N, Maksagak B, Milton M I, Ford J D, Hawley N L, Dubrow R (2021). Determinants, effects, and coping strategies for low-yield periods of harvest: a qualitative study in two communities in Nunavut, Canada. Food Security.

[erfsadf5c9bib18] Government of Canada (2018a). Aboriginal Peoples Survey–Nunavut Inuit Supplement, 2017. https://www150.statcan.gc.ca/n1/daily-quotidien/181126/dq181126d-eng.htm.

[erfsadf5c9bib19] Government of Canada (2018b). Labour Market Experiences of Inuit: Key Findings from the 2017 Aboriginal Peoples Survey. https://www150.statcan.gc.ca/n1/pub/89-653-x/89-653-x2018004-eng.htm.

[erfsadf5c9bib20] Government of Nunavut (2012). Chronic Disease Summary: Nunavut (Fiscal Years 2004‐2012). https://www.gov.nu.ca/sites/default/files/chronic_disease_fy2004-2012_final.pdf.

[erfsadf5c9bib21] Government of Nunavut (2014). Health Profile Nunavut: Information to 2014. https://www.gov.nu.ca/sites/default/files/files/health_profile_nunavut.pdf.

[erfsadf5c9bib22] Harder M T, Wenzel G W (2012). Inuit subsistence, social economy and food security in Clyde River, Nunavut. Arctic.

[erfsadf5c9bib23] Hillemann F, Beheim B A, Ready E (2023). Socio-economic predictors of Inuit hunting choices and their implications for climate change adaptation. Phil. Trans. R. Soc. B.

[erfsadf5c9bib24] Inuit Tapiriit Kanatami (2019). National Inuit Climate Change Strategy. https://repository.oceanbestpractices.org/handle/11329/1944.

[erfsadf5c9bib25] Kenny T-A, Hu X F, Kuhnlein H V, Wesche S D, Chan H M (2018). Dietary sources of energy and nutrients in the contemporary diet of Inuit adults: results from the 2007–08 Inuit Health Survey. Public Health Nutr..

[erfsadf5c9bib26] Kontopantelis E, Reeves D (2010). Metaan: random-effects meta-analysis. Stata J..

[erfsadf5c9bib27] Kuhnlein H V, Receveur O (2007). Local cultural animal food contributes high levels of nutrients for Arctic Canadian Indigenous adults and children. J. Nutr..

[erfsadf5c9bib28] Kuhnlein H V, Receveur O, Soueida R, Egeland G M (2004). Arctic indigenous peoples experience the nutrition transition with changing dietary patterns and obesity. J. Nutr..

[erfsadf5c9bib29] Ledrou I, Gervais J (2005). Food insecurity. Health Rep..

[erfsadf5c9bib30] Little M, Hagar H, Zivot C, Dodd W, Skinner K, Kenny T-A, Caughey A, Gaupholm J, Lemire M (2021). Drivers and health implications of the dietary transition among Inuit in the Canadian Arctic: a scoping review. Public Health Nutr..

[erfsadf5c9bib31] Naylor A W, Ford J D, Pearce T, Fawcett D, Clark D, van Alstine J (2021). Monitoring the dynamic vulnerability of an Arctic subsistence food system to climate change: the case of Ulukhaktok, NT. PLoS One.

[erfsadf5c9bib32] Naylor J (2019). Assessing the Effect of Food Retail Subsidies on the Price of Food in Remote Indigenous Communities: a Case Study of the Nutrition North Canada Subsidy Program. https://atrium.lib.uoguelph.ca/xmlui/handle/10214/16966.

[erfsadf5c9bib33] Nichols A (2010). Regression for Nonnegative Skewed Dependent Variables. https://www.stata.com/meeting/boston10/boston10_nichols.pdf.

[erfsadf5c9bib34] Nunavut Planning Commission (2016). 2016 Draft Nunavut Land Use Plan. https://www.nunavut.ca/sites/default/files/2016_draft_nunavut_land_use_plan_0.pdf.

[erfsadf5c9bib35] Nunavut Planning Commission (2021). 2021 Draft Nunavut Land Use Plan. https://www.nunavut.ca/sites/default/files/21-001e-2021-07-08-2021_draft_nunavut_land_use_plan-english.pdf.

[erfsadf5c9bib36] Pearce T, Ford J, Willox A C, Smit B (2015). Inuit traditional ecological knowledge (TEK), subsistence hunting and adaptation to climate change in the Canadian Arctic. Arctic.

[erfsadf5c9bib37] Petimar J (2022). Assessment of calories purchased after calorie labeling of prepared foods in a large supermarket chain. JAMA Intern. Med..

[erfsadf5c9bib38] Priest H, Usher P J (2004). The Nunavut Wildlife Harvest Study, August 2004.

[erfsadf5c9bib39] Qikiqtani Inuit Association (2018). Evaluating the Role of Marine-Based Harvesting in Food Security in the Eastern Arctic. https://www.nirb.ca/publications/strategic%20environmental%20assessment/190125-17SN034-QIA%20Report%20Re%20Marine%20Based%20Harvesting-IEDE.pdf.

[erfsadf5c9bib40] Ready E (2016). Challenges in the assessment of Inuit food security. Arctic.

[erfsadf5c9bib41] Sharma S, Cao X, Roache C, Buchan A, Reid R, Gittelsohn J (2010). Assessing dietary intake in a population undergoing a rapid transition in diet and lifestyle: the Arctic Inuit in Nunavut, Canada. Br. J. Nutr..

[erfsadf5c9bib42] Sheehy T, Roache C, Sharma S (2013). Eating habits of a population undergoing a rapid dietary transition: portion sizes of traditional and non-traditional foods and beverages consumed by Inuit adults in Nunavut, Canada. Nutr. J..

[erfsadf5c9bib43] Sheikh N, Egeland G M, Johnson-Down L, Kuhnlein H V (2011). Changing dietary patterns and body mass index over time in Canadian Inuit communities. Int. J. Circumpolar Health.

[erfsadf5c9bib44] Statham S (2012). Inuit food security: vulnerability of the traditional food system to climatic extremes during winter 2010/2011 in Iqaluit, Nunavut. https://escholarship.mcgill.ca/concern/theses/6682x7587.

[erfsadf5c9bib45] Statham S, Ford J, Berrang-Ford L, Lardeau M-P, Gough W, Siewierski R (2015). Anomalous climatic conditions during winter 2010–2011 and vulnerability of the traditional Inuit food system in Iqaluit. Nunavut. Polar Rec..

[erfsadf5c9bib46] Statistics Canada (2014). Health Trends, Nunavut. https://www12.statcan.gc.ca/health-sante/82-213/Op1.cfm?Lang=eng%252626TABID=0%252626IND=R%252626SX=TOTAL%252626PROFILE_ID=0%252626PRCODE=62%252626change=no.

[erfsadf5c9bib47] Statistics Canada (2018). Household Spending in the Territorial Capitals, 2017. https://www150.statcan.gc.ca/n1/pub/11-627-m/11-627-m2018052-eng.htm.

[erfsadf5c9bib48] Statistics Canada (2019). Census Profile, 2016 Census: Territorial Data. https://www12.statcan.gc.ca/census-recensement/2016/dp-pd/prof/search-recherche/lst/results-resultats.cfm?Lang=E%252626TABID=1%252626G=1%252626Geo1=PR%252626Code1=62%252626Geo2=PR%252626Code2=01%252626GEOCODE=62%252626type=0.

[erfsadf5c9bib49] Statistics Canada (2024). Census Profile, 2021 Census of Population. https://www12.statcan.gc.ca/census-recensement/2021/dp-pd/prof/search-recherche/lst/results-resultats.cfm?Lang=E%252626GEOCODE=62.

[erfsadf5c9bib50] Tarasuk V, Mitchell A, Dachner N (2013). Household Food Insecurity in Canada, 2011. https://proof.utoronto.ca/wp-content/uploads/2014/01/foodinsecurity2011_final.pdf.

[erfsadf5c9bib51] The Expert Panel on the State of Knowledge of Food Security in Northern Canada (2014). Aboriginal Food Security in Northern Canada: an Assessment of the State of Knowledge: the Expert Panel on the State of Knowledge of Food Security in Northern Canada. https://cca-reports.ca/wp-content/uploads/2018/10/foodsecurity_execsummen.pdf.

[erfsadf5c9bib52] Tukey J W (1962). The future of data analysis. Ann. Math. Stat..

[erfsadf5c9bib53] Wakegijig J, Osborne G, Statham S, Issaluk M D (2013). Collaborating toward improving food security in Nunavut. Int. J. Circumpolar Health.

[erfsadf5c9bib54] Warltier D W, Landry-Cuerrier M, Humphries M M, Giguère N (2021). Valuation of country food in Nunavut based on energy and protein replacement. Arctic.

[erfsadf5c9bib55] Wenzel G W (2000). Sharing, money and modern Inuit subsistence: obligation and reciprocity at Clyde River, Nunavut. Senri Ethnol. Stud..

[erfsadf5c9bib56] Wenzel G W (2013). Inuit and modern hunter-gatherer subsistence. Études/Inuit/Studies.

[erfsadf5c9bib57] Wenzel G W (2017). Canadian Inuit subsistence: antinomies of the mixed economy. Hunter Gatherer Res..

[erfsadf5c9bib58] Wooldridge J M (2010). Econometric Analysis of Cross Section and Panel Data.

